# Simulation Training in Psychiatry for Medical Education: A Review

**DOI:** 10.3389/fpsyt.2021.658967

**Published:** 2021-05-21

**Authors:** Marie-Aude Piot, Chris Attoe, Gregoire Billon, Sean Cross, Jan-Joost Rethans, Bruno Falissard

**Affiliations:** ^1^School of Medicine, Faculty of Health, University of Paris, Paris, France; ^2^Department of Psychiatry, Institute Mutualiste Montsouris, Paris, France; ^3^Epidemiological and Public Health Research Centre, Villejuif, France; ^4^Maudsley Simulation, South London and Maudsley National Health Service (NHS) Foundation Trust, London, United Kingdom; ^5^Skillslab, Faculty of Health, Medicine and Life Sciences, Maastricht University, Maastricht, Netherlands; ^6^Department of Public Health, School of Medicine, University Paris Saclay, Villejuif, France

**Keywords:** simulation training, mental health, learning, patient simulation, education medical

## Abstract

Despite recognised benefits of Simulation-Based Education (SBE) in healthcare, specific adaptations required within psychiatry have slowed its adoption. This article aims to discuss conceptual and practical features of SBE in psychiatry that may support or limit its development, so as to encourage clinicians and educators to consider the implementation of SBE in their practice. SBE took off with the aviation industry and has been steadily adopted in clinical education, alongside role play and patient educators, across many medical specialities. Concurrently, healthcare has shifted towards patient-centred approaches and clinical education has recognised the importance of reflective learning and teaching centred on learners' experiences. SBE is particularly well-suited to promoting a holistic approach to care, reflective learning, emotional awareness in interactions and learning, cognitive reframing, and co-construction of knowledge. These features present an opportunity to enhance education throughout the healthcare workforce, and align particularly well to psychiatric education, where interpersonal and relational dimensions are at the core of clinical skills. Additionally, SBE provides a strategic opportunity for people with lived experience of mental disorders to be directly involved in clinical education. However, tenacious controversies have questioned the adequacy of SBE in the psychiatric field, possibly limiting its adoption. The ability of simulated patients (SPs) to portray complex and contradictory cognitive, psychological and emotional states has been questioned. The validity of SBE to develop a genuine empathetic understanding of patients, to facilitate a comprehensive multiaxial diagnostic formulation, or to develop flexible interpersonal skills has been criticised. Finally, SBE's relevance to developing complex psychotherapeutic skills is much debated, while issues such as symptom induction in SPs or patients involvement raise ethical dilemmas. These controversies can be addressed through adequate evidence, robust learning design, and high standards of practice. Well-designed simulated scenarios can promote a positive consideration of mental disorders and complex clinical skills. Shared guidelines and scenario libraries for simulation can be developed, with expert psychiatrists, patients and students involvement, to offer SPs and educators a solid foundation to develop training. Beyond scenario design, the nuances and complexities in mental healthcare are also duly acknowledged during the debriefing phases, providing a crucial opportunity to reflect on complex interpersonal skills or the role of emotions in clinicians' behaviour. Considered recruitment and support of SPs by clinical educators can help to maintain psychological safety and manage ethical issues. The holistic and reflexive nature of SBE aligns to the rich humanistic tradition nurtured within psychiatry and medicine, presenting the opportunity to expand the use of SBE to support a range of clinical skills and workforce competencies required in psychiatry.

## Introduction

Since the first experimentations with simulators in medicine in the sixties, simulation-based education (SBE) has become a well-established method to bridge the gap between theory and practice in medical education, moving from “best secret” to “best practice” (1). SBE is now recognised as an effective training tool to enhance medical error management, patient care and safety, and health professional team training (2). This training is defined as “a technique that creates a situation or environment to allow persons to experience a representation of a real event for the purpose of practice, learning, evaluation, testing, or to gain understanding of systems or human actions” (3).

SBE appears to be a valuable and multidimensional method to improve healthcare delivery, deserving further development and adoption. In psychiatry, despite a strong history of utilising role play in the teaching of psychotherapy and in nursing education (4), its development has remained limited. This suggests that specific considerations and adaptations of SBE might be required to allow for its integration into routine mental health education.

This article aims to discuss conceptual and practical features of SBE in psychiatry that may support or limit its development, so as to encourage clinicians and educators to consider the implementation of SBE in their practice.

We based our argument on two main sources. To document SBE principles and the context of its development, we searched for the main historical texts which supported SBE development and the recognised guidelines to implement SBE based on updated evidence. Then, we focussed on the specific literature on SBE in psychiatry, building on a wide systematic review previously carried out, the protocol of which has been reported elsewhere (5). Furthermore, the content of this article reflects the practical experiences of psychiatrists and multi-professional teams working in clinical education and SBE for a number of years.

The article structures the argument through five distinct sections. We first provide the historical context which shaped the development of SBE in medicine and psychiatry. Subsequently, we outline some key theoretical frameworks underpinning SBE in mental health, and practical features of its implementation. Against this background, we discuss the opportunities and suitability of harnessing SBE more routinely within psychiatry to improve education, professional development, and clinical practice. We then present some of the controversies surrounding simulated practice in psychiatry, which may limit its development and require careful consideration. Finally, we discuss possible strategies and approaches to address these issues and capitalise upon the opportunities and broad potential offered by SBE in psychiatry.

## Historical Context

Critical developments on SBE first happened in sectors liable to a high degree of risk, such as aviation, aeronautics and nuclear safety, to reduce human errors in high stake context. In healthcare, emergency medicine and intensive care were the first to thoroughly adopt this pedagogical tool. These fields are shaped by a culture of risk management, requiring a high degree of technical and procedural control based on rigorous teamwork. It became apparent that genuine attempts to minimise risks would need to tackle the “human factors” (6). What required improvement were “non-technical skills,” including personal features (stress, tiredness), cognitive skills (situational awareness, decision making and planning) and social abilities (communication, teamwork and leadership). The field of human factors has had a considerable influence in the development of SBE, bringing back the ancient proverb “*Errare humanum est*” to the forefront of medical education and spurring a growing body of literature on cognitive bias (7).

While several countries conducted studies to assess the health and cost impact of medical errors (8), the American psychologist James Reason developed models to retrospectively analyse errors and enhance work practices. Reason offered a broad perspective, including both an analysis of the linear causal chain driving medical error, and a systemic analysis of the context in which the error happened. In the aviation industry, this led to the development of “Crew Resource Management” training, which has remained a robust framework for interprofessional training in healthcare (9), and contributed to the shift in focus from individual to collective care and learning.

Another strand leading to the development of SBE can be traced back to early experiments to emulate pathological states. In 1963, the neurologist Howard Barrow in Los Angeles conducted the first systematic experiments to portray neurological syndromes through acting, initially simulating multiple sclerosis (10). Here was born a new partner in clinical education: the simulated patient (SP). Despite early controversies about the cost and feasibility of using actors to play patients, SPs were rapidly adopted in medical education in the USA as powerful allies in training. They were made to portray an ever-growing range of physical symptoms and challenges of the doctor-patient relationship, and took part in clinicians' assessments, with the progressive generalisation of Objective Structured Clinical Examinations (OSCEs; see Table 1).

**Table 1:** Simulation technologies.

**Technology**	**Definition**	**Applications, especially in psychiatry**
Human simulation:	A “methodology that involves human role players interacting with learners in a wide range of experiential learning and assessment contexts” (11).	
• Role play	The patient role-player is “asked to be someone quite different from themselves and, with little or no preparation, perform in front of peers and teachers” (12).	Role-playing are usually reported as appropriate for mental disorders less difficult to portray by a novice (as typical depression, or some drug abuse disorders) (13).
• Simulated patient	“A person who has been carefully coached to simulate an actual patient so accurately that the simulation cannot be detected by a skilled clinician. In performing the simulation, the SP presents the gestalt of the patient being simulated; not just the history, but the body language, the physical findings, and the emotional and personality characteristics as well” (11).	Conversely for complex portrayals – such as schizophrenia or mania – for novice trainees can create the risk for providing caricatures or superficial simulations. SPs do enhance the validity of simulations however, including for all other disorders. Digital libraries of videos for medical education could improve this validity, as reported in recent articles (14–16). The most recent and exhaustive guidelines on SP training were built by the Association of Standardized Patient Educators (ASPE), upon the principles of safety, quality, professionalism, accountability and collaboration (11).
• Standardized patients	It means highly replicable scenario and SP training-, often used in high stakes educational decision to improve fidelity, enabling equity between the learners.	It is often used in high stakes educational decision – as OSCEs - to improve fidelity, enabling equity between the learners (17).
Manikin	“Full or partial body simulators that can have varying levels of physiologic function and fidelity” (3).	The use of manikins to recreate patients is more devoted to medical specialties where procedural simulation (and its high level of technic) is the priority, and the reproduction of non-technical features – as non-verbal signs of emotions- less important. However in psychiatry, some specific area may benefit from manikin, such as training discrete procedural skills as Electroconvulsivo-therapy (18, 19).
Virtual reality:	“The use of computer technology to create an interactive three-dimensional world in which the objects have a sense of spatial presence” (3) with which an individual can actively interact.	Its emerging went with important efforts to make encounters with virtual patients realistic enough to effectively engage learners. Studies suggest that VR have an impact on communication, teamwork and decision-making (20). Given the complexity in psychiatry, its use may improve self-confidence (21), work on assumption and believes toward patients and focus on clinical reasoning before meeting with a human SP. The opportunity to repeat the scenarios as many time as wished and for some of them, to easily broadcast in personal tables and smartphone, may recoup the cost of the high initial funding required to design appropriate VR, while extending infinitely the dissemination.
• e.g.,: Voice simulation	The “use of sounds and voice through an electronic medium to portray the sounds encountered by a schizophrenic patient” (22).	Designed by patients themselves - inside the movement of patient experiential recovery, as Patricia Deegan - this technology enables the health trainee to experiment in part auditory hallucinations from a first-person view. Trainees are often missioned to complete cognitive tasks during the listening, to increase the proximity with real schizophrenic experiences and their struggles for completing life challenges. Through improving the identification with patients, this simulation experience increases the empathy toward people with schizophrenia (23), while reducing previous assumptions and believes that often stigmatize person with mental disorders (24).
Objective structured clinical exams (OSCEs)	OSCE is composed by series of short stations that the trainee has to complete, each of them focusing on one clinical or other professional task; examination is performed through direct observation, checklist, scale, learner presentation or written follow-up exercise (3).	An exhaustive guide has been developed by the Psychiatric Skills Assessment Project (PSAP) of University of Toronto (17) and updated since and updated since (25), describing of several steps to implement different psychiatric scenarios in an OSCE.

Over the period, SPs were experimentally introduced in psychiatric institutions with a more radical aim in mind. In the famous experiment led by Rosenhan, undercover SPs portraying auditory hallucinations provided empirical support to the radical claims of the anti-psychiatry movement that psychiatric disorders were primarily a social construct perpetuated by its institutions (26). The undercover SPs remained hospitalised for 7 up to 52 days, despite dropping their symptoms following their admission as in-patients! This experiment highlighted the worryingly pervasive impact of some diagnostic labels, at times muddling clinical reasoning in psychiatry, while challenging the relevance of established boundaries between reason and insanity. While these challenges might have contributed to the more positivist approach to classify mental health disorders in psychiatry, from the third revision of the Diagnostic and Statistical Manual (DSM III, 1980) onwards, Rosenhan's experiment is a striking example of the power of simulation to trigger deep reflexivity on the complexity of psychiatric practices.

Several efforts were devoted to improving SP-based pedagogy, particularly through the creation of several associations, of which the Association of Standardized Patient Educators (ASPE) in 2001 is the most well-known. ASPE aims to foster advances in SP-based pedagogy, assessment, research, and scholarship (11). ASPE became a key player in human simulation, exemplifying the horizontal and democratic culture of SBE. By gathering both educators, real patients and SPs to define SP use guidelines, ASPE supports patients' influence in medical education, and ultimately medical care.

## Framework

### Theories

The development of SBE is firmly grounded in several key developments of adult learning theories (27). Behaviourism helped to consider how pedagogical conditions support or limit technical acquisition (28). Cognitivism informed instructors on the perception and processing of information, recognizing further the essential role of emotions, motivation and metacognitions in learning (29). The notion of “self-efficacy” developed in Bandura's social cognitive learning theory was especially important for defining the belief in one's capacity to take action as a crucial driver of performance (30). The experiential learning theory developed by David Kolb and often summarised in the figure of a learning cycle also deeply influenced SBE (31, 32). Social constructionism has strongly influenced SBE, through the notion of subjective and collective construction of meaning, and the role of community and social context in learning (33–36). Other approaches, such as Cultural-Historical Activity Theory, further emphasised historical and cultural contexts that shape the group elaboration of meaning (37), while others still have helped to describe how SBE can challenge participants' assumptions and beliefs towards patients with mental disorders, for example transformative learning theory (38).

Finally, SBE has borrowed beyond the traditional boundaries of medical education. Psychodrama exposed mental health workers to the deep transformations instigated by simulations with the therapist and other patients, beyond one-to-one consultation (39). Significantly, SPs' performances share common features with acting and theatre practice. The notion of “state of I am,” as developed by the theatre practitioner Constantin Stanislavsky, may be relevant for both of these contexts; that is “the point where I begin to feel myself in the thick of things, where I begin to coalesce with all the circumstances suggested by the playwright” (40). Theatre has also been brought to medical education to support students' empowerment in their learning and professional developments, as well as a means to question the social transformations of health systems and the very structure of medical care. It remains important for the field of SBE to continue to refine and develop its pedagogical approach, while making these underpinnings explicit to both educators and learners.

### Practical Implementation

SBE starts with defining the issues to address, the learners' needs and specific learning objectives. Scenarios are carefully designed to portray either common psychopathological presentations, for basic training, or increasingly atypical or challenging situations for advanced learners (35).

SBE encompasses a diverse array of technologies developed to recreate clinical situations, from simple role play, to high-fidelity manikins and human simulated patients, to complex virtual reality (VR). Table 1 summarizes SBE techniques, including a focus on those with a particular relevance to psychiatry.

**Figure 1:**
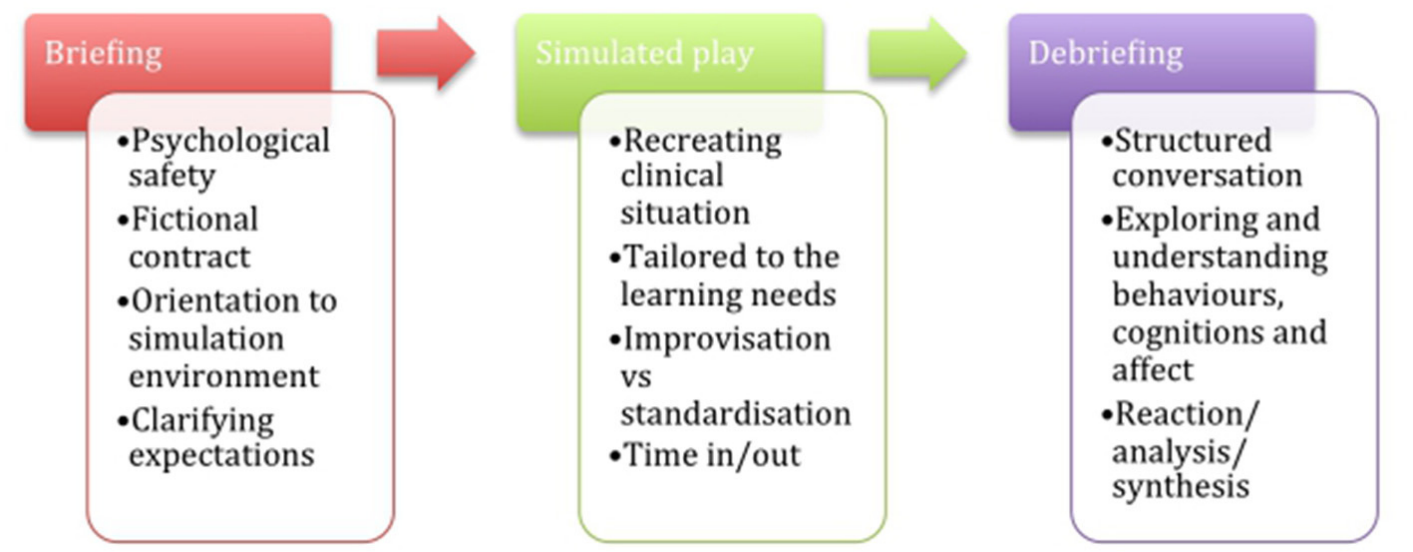
The basic three stages process of SBE.

Single episodes of SBE typically include three practical stages (Figure 1), with aims and remits varying according to participant groups and learning objectives. The first phase is the “pre-simulation briefing,” or pre-briefing. This is an essential step to establish the safe learning environment required for SBE and prime learners for the intended learning activities (41). The second phase is the simulated scenario itself. It aims to recreate realistic clinical situations to embed participants in with sufficient fidelity and alignment to specified learning objectives. The third stage is the debriefing, where facilitated reflective conversations aim to convert the simulation experience and reflection into learning (42). Different debrief models can be used along a continuum from direct feedback and instructional teaching to more facilitative and reflective approaches (43, 44). There are consensual guidelines on some key features of debriefing in SBE's instructional design (45): the psychological safety of participants; their active involvement; Socratic questioning instead of direct feedback; led by a facilitator with specific training in debriefing; and who announces and enacts respect for the learners as a competent human being willing to improve, referred to as the basic assumption or principle. Other points remain debated, such as observers' roles in improving learning (46) or the place of video feedback (44).

## Opportunities and Suitability of SBE in Psychiatry

### Person-centred Care

The “person-centred medicine” (PCM) movement developed the explicit promotion of the subjectivity of the person with an illness and including the sociocultural environment in care delivery (47). The World Psychiatric Association supported the development of this approach in psychiatry affirming “*the whole person of the patient in context* as the centre and goal of clinical care and health promotion,” within a multidimensional approach (including the biological, psychological, sociocultural, spiritual, ethical and artistic dimensions, as well as the users' views) (48).

Calling for skilled practitioners who can integrate an interactional and personalised model of care, PCM challenges traditional educational approaches (49). Authoritarian and directive teaching, overlooking the learner's experience, matches reductionist and paternalistic models of care overlooking the patient's experience (49). Consequently, PCM requires an educational approach that promotes an interactive and dialectical learning environment. Such educational environments encourage participants to include their personal background and motivations in the learning process, and make the development of a reflective understanding a cornerstone to becoming a competent and self-aware practitioner. SBE offers several practical features which align with PCM's agenda, some of which are particularly relevant to psychiatry where complex subjective and interpersonal processes are at the heart of the delivery of care.

By the virtue of its experiential nature, SBE involves the “whole person” of both the learner and the patient. Contrary to traditional teaching, such as large group lectures set up for knowledge acquisition, SBE allows to work at the same time on the physical, affective, and cognitive features involved in the simulated scenario. SBE provides a unique opportunity to rehearse and enact the complex interpersonal processes pertaining to psychiatric care. During the simulated encounter, the person played by the SP presents themselves “as a whole,” instead of being first considered as a collection of symptoms, as a psychiatric handbook may suggest. These initial perceptions, the “first impressions” with the patient, were previously emphasised by psychiatric phenomenology as a global perception essential to understand the patient through pattern recognition (50, 51). In addition, several scenarios can be played successively to portray the patient journey through clinical pathways over a period of time. SBE can support a diachronic understanding of the patient, which provides a more comprehensive perspective on the patient than the snapshot view of a diagnosis or a single presentation.

Psychiatric SBE can be construed as a learner-centred approach, further promoting a person-centred approach in clinical care. The flexibility of SP and trainers allow adaptation of the experience, tailored feedback and reflection to the needs and characteristics of the learners. By including physical, affective and cognitive features in the learning process, participants are also considered as a “whole person.” This is particularly relevant when training some essential psychiatric skills, for instance the empathetic skills that learners should demonstrate towards each other and their patients. Indeed, this can even extend to learning experiences that allow clinicians to reflect on their own health or work-related needs.

Finally, SBE offers an increasingly recognised role to real and expert patients with mental disorders to get involved in clinical education and have a say in the way they want to be cared for. Indeed, SBE can include patients at each step of the SBE developments, including scenario design, SP training, simulated scenarios, and feedback in debriefing.

### The Nature of Psychiatric Care

SBE is particularly suitable to psychiatric care as evidenced by its facilitation of a person-centred approach, the acquisition of reflective skills, a common focus on attitudinal change and multi-disciplinary work, and cognitive reframing and co-construction of care. Recently, learner-centred approaches have highlighted current issues encountered by clinicians – such as racial tensions or transphobia – to co-construct simulations in real time together with clinicians, trained simulated patients and educators (35, 36). These factors align to the cognitive and interpersonal skills required for psychiatric care and allow education to remain up to date with clinical practices and issues encountered.

Prevailing changes within healthcare further highlight the opportunity afforded by SBE, such as reduced availability of patient contact and bedside teaching in clinical placements due to evolutions in psychiatric care delivery, for example ward closures, community psychiatric service restructure, specialist teams, fewer senior clinicians, and more severe and complex patients (52). It is then surprising that SBE has remained relatively underdeveloped in the psychiatric field compared to other specialities, and that psychiatry has not led the development of SBE in areas with such complex learning outcomes to push the boundaries of simulated patient scenarios (4).

### Reflective Practice and Attitudinal Change

The opportunity to acquire or deepen reflexive skills may be especially relevant in psychiatry, where health workers typically need these skills to develop a treatment plan, rather than primarily applying structured guidelines. Likewise, the opportunity to reflect collectively on the diversity of perceptions for the same clinical situation mirrors the way that psychiatric teams collectively build these representations of the patients. The opportunity to challenge assumptions and beliefs of learners should be welcomed in psychiatry, prioritising self-awareness and reflection. Assumptions, beliefs, and attitudes require challenging throughout psychiatric practice and consequently throughout psychiatric education at all levels for all professionals.

Indeed, when patients' presenting complaints are “psychiatric behaviour disorders,” a label of “manipulative,” or “borderline personality,” or with a “history of drug abuse,” patients are often met with negative assumptions by practitioners (53). Many health students have negative attitudes towards this discipline as individuals with mental disorders are often feared, stigmatised, and stereotyped (54–56). They are often seen as difficult to understand, even hermetic, with students reporting they often don't know what to say or worrying that they might cause harm by acting in the wrong way, due to the complexity of relational skills and attitudes required (57). Even newly qualified doctors lack confidence in assessing and managing common psychiatric problems more than other conditions (52). For adolescent SPs portraying psychiatric conditions, a study found increased anticipated role discomfort because of stigma related with mental disorders (58).

Considering dual-process theory on clinical reasoning, these negative feelings are bound to influence the intuitive response of health workers and impact on the reliability of their clinical reasoning (53). Conversely, attitudes relating to self-confidence, anxiety, assumptions and beliefs are common outcomes reported in psychiatric SBE research (13), emphasising the great effort ensured by SBE to make psychiatry more accessible to medical students and health workers more globally. This may support fighting against stigmatisation described more than 40 years ago (59) and still present even among health care workers.

### Multi-disciplinary Team Working

SBE supports the long tradition of multidisciplinary teams working in psychiatry required to provide high quality care for mental disorders. The ability of SBE to bring together different professions and specialties to learn together and in multi-disciplinary teams reflects how care is and should be delivered (4). When clinical teams engage in SBE together, this creates opportunities to gain insights into their work as a team in a dedicated educational space free from clinical demands, supporting creativity and translation of learning into practice. When health workers engage in interprofessional SBE with unfamiliar colleagues, their individual development and learning from others is complementary across the group. Further, SBE can expose health workers to unusual or uncommon scenarios that they may have had limited opportunity to engage with individually or within teams. This is particularly true for more inexperienced staff, and for emergency or out-of-hours situations, such as managing a mental health crisis (60).

### Emerging Evidence on Psychiatric SBE

Outcomes of RCTs, non-RCTs, and pre/post-test studies provide some evidence on the effectiveness of SBE in psychiatry, collated in a recent systematic review including 163 studies (13). Two third of studies included attitude outcomes, one-third included skills and knowledge, while behaviours and patients' benefits were included in 10% of identified studies. In the 27 RCTs included in meta-analysis, significant differences were found at immediate post-tests for simulation groups compared with both active and inactive control groups on attitudes, skills, knowledge and behaviours of medical doctors and participants. Significant differences were also found at 3-month follow-ups with large effect sizes for behaviour-based outcomes and small effect sizes for skills-based and patient benefits outcomes. Moreover, two third of pre/post-test studies found significant differences on attitudes, skills, knowledge and behaviours of participants, alongside around half of the controlled studies. However, the low number of controlled studies undermine the strength of the evidence. Similarly, regarding patients' benefits, the smaller number of studies and the heterogeneity amongst the time-points of assessment make interpretation difficult. The authors concluded that the number of RCTs was sufficient for pooling meta-analyses, but not enough to provide overwhelming evidence, despite some very high quality research (61). They encourage further research including RCTs, focused on participants' behaviours and patient outcomes, longitudinal evaluations, and even long-term assessment of cost-effectiveness.

The review highlighted high heterogeneity across studies, including pedagogical conditions (e.g., scenarios, debriefing modalities, length, educational aims, adjuvant pedagogies in more than three questers of studies), participant levels (mixing medical levels and/or health professions), and the outcomes and instruments to measure them. While this heterogeneity may limit the quality of the evidence, it is consistent with the diverse and complex nature of psychiatry and the multifaceted nature of SBE, increasing the external validity of these results.

## Controversies in Psychiatric SBE

### Intensity and Requirements of SBE

SBE requires deep, intense involvement and engagement for both participants and trainers. For participants, performing in front of peers can be demanding. Unpredictable simulated scenarios can reveal intimate parts of oneself and one's behaviour, such as spontaneous emotions or reactions that are usually privately shared with the patient or with familiar colleagues. Thus, the high cognitive, emotional, and physical load for participants while peers and trainers are observing, can generate stress, performance anxiety, or fears of being judged. This stress could inhibit participants' interest in SBE and their ability to derive learning from all aspects of the training experience. Additionally, feedback or collective discussion on features with which the participant is not familiar may be perceived as intrusive or uncomfortable without an appropriately safe and collaborative learning environment. Further, debriefing that aims to uncover and maybe reframe emotions and cognitions can cause discomfort due to exposure within the group. However, it must be noted that the power and potential of SBE connects closely with the opportunity to create a learning environment that facilitates the friendly challenge and constructive discomfort that allows the generation of deep and complex learning. The key to ensuring this opportunity lies with facilitators to prepare and manage interactions with and within the group accordingly, although SBE literature has struggled to provide clear and thorough guidance (42, 45).

For trainers too the implementation of SBE may be very challenging, requiring time and effort to achieve high quality. The design of well-suited scenarios and guidance for simulated patients require careful consideration, piloting, and continual adjustments to tailor to the learners' needs. The recruitment, training, support, and monitoring of SPs are time-consuming and require continuous work and consideration of the SP as an important member of the training team. Trainers themselves must be trained and supported both formally and informally, reviewing and encouraging their development and reflection. Further, the efforts for trainers on the day begin and end well before and after the training has been completed. Indeed, the time requirements can often lead trainers to opt for shorter SBE formats using directive feedback and simpler scenarios. The intensive small group format may also be prohibitive for systems, settings, and countries where the ratio of trainers to participants is low or other resources are lacking beyond time and human capacity. Even technologically advanced SBE, such as virtual reality, requires considerable time and resource demands in creating and testing realistic virtual scenarios.

Consequently, the requirements of SBE for participants, trainers, and systems are considerable. This may explain why role play has been used for a long time in psychiatry. However, beyond time and cost often mentioned as a barrier to SBE, its implementation in psychiatry has further specific challenges.

### Specific Challenges of SBE in Psychiatry

Some authors still question the ability of SPs to embody a complex set of often contradictory cognitive, psychological and emotional features to support valid learning (12). This raises necessary distinction between an authentic – the “impossibility to distinguish SPs from patients” (62)– and a valid portrayal. Authenticity supports the learner involvement in the learning, since students can report difficulties engaging with the scenario when they perceive the simulation as unrealistic (62). However, the “impossibility to distinguish” refers to the rater's subjective perception, possibly restrained by a limited experience for most students. Thus, a slightly too caricatured portrayal missing some ambivalent and conflicting features may appear authentic to a student, but not to an experienced psychiatrist.

Another risk, if SPs were unable to elicit a learner's empathy, would be to paradoxically lead to a shallow interaction that prevents participants from detecting nuances and subtlety in the diagnosis. This may affect the validity of training, while inducing superficial or even inadequate representations. As a result, there is a specific need for realism and an emotionally engaging depiction of patients with mental disorders in psychiatric SBE, to enable learners to develop a real understanding of the patient experience, a comprehensive multiaxial differential diagnosis, and a flexible relationship. However, conversely, teaching psychotherapeutic and complex interpersonal skills raises other issues regarding emotions. Indeed, there may be a difference between an empathic response (of the learner) to an actual dramatic character (the SP) compared with the response they would have to a future real patient whom the learner will support (63). While SPs are trained to arouse emotion, patients with mental disorders do not plan how they will present to the physician. The part of themselves to be uncovered may be uncertain, even resistant, as much for themselves as for the clinician. The role of the physician is precisely to establish an authentic empathic rapport to help the patient to soften conflicted feelings, by “feeling ahead” of the patient and intuiting what can't be linked (63). These essential psychotherapeutic skills may remain elusive to the SBE set up.

Concurrently, the notion of prototypes of clinical portrays, often used to offer appropriate training to novices, may be invalid in psychiatry due to the singular experience of each patient with a mental disorder. This issue is also raised by recent developments in virtual reality (64, 65), interviewing a patient with mental disorders in Second Life (66), or an adolescent with PTSD (67), which question even more the believability of virtual characters, and their ability to elicit a realistic experiences for novice clinicians. Here there is a risk that participants learn a reality about patient with mental disorders that is inaccurate, based on portrayals of these experiences rather than real experiences themselves, such as interacting with real patients. This has been described as “hyper-reality” (68), where an excessive use of symbols substitutes the real experience, first highlighted by mass media in the seventies. Yet, beyond false psychopathological features, the difference between learner/fictional character relationship and the real doctor-patient relationship would enable students to act out good relationships without being authentically involved (12).

In addition, the choice of mental disorders pictured is often driven by epidemiological considerations, especially for early career training. This creates the risk of reinforcing stigmatisation and stereotypes towards patients. For example, characterizing an eating disorders patient as a white middle class female. There is a constant balance between a realistic portrayal of individual, social, and epidemiological experience of mental disorder and unhelpful stereotyping. This requires the involvement of real patients in education and reflexivity from the trainers and SPs.

Moreover, while promising results are reported for SPs in psychiatric OSCE assessment, there is a trade-off between the standardisation process required and the validity of the psychiatric portrayal (69). Indeed, exams require a strong reliability, both test-retest and inter-rater reliability, in scenarios in order to offer equal opportunities in assessment for learners. At the same time, the complexity of psychiatric care sometimes needs a lot of flexibility to be valid in simulated scenarios, according a learner's reaction. It may mean that SPs should often both reflect the patient they are portraying, and their own personal response to the psychopathology being presented, to remain plausible (12). More globally, this raises the underlying paradox inherent to psychiatric presentations: there is a direct conflict between a rigidly scripted portrayal and a valid and realistic portrayal, which requires flexible adaptation to an unfolding interaction.

Furthermore, there are areas of psychopathology where the fictional nature of the simulation set up might create some confusion and somehow limit simulation's educational benefits. Pretend mode, false beliefs and the blurring of boundaries between fiction and reality are all common features of simulation practice as well as a number of psychiatric disorders, e.g., “as-if personalities,” malingering, narcissistic disorders. The practitioner has to uncover the part of the person, which may be either simulated (consciously or not), factitious, mythomaniac, delusional, etc. In these cases, with the fictional nature of the simulation set-up may confuse the matter further and limits effective training (63), while learners need a secure well-defined frame to develop reflective practice.

Finally, because of the complexity of mental disorder experience, the ability of SPs to provide feedback on the phenomenological experience of psychosis, for example, may be more complex and unfamiliar than for a SP who portrays diabetes. A lack of nuance creates the risk to perpetuate stigma, albeit unwillingly, through inappropriate feedback and portrayal of illness.

Given the above issues of validity and complexity, SP training in psychiatry requires careful consideration, from rigorous recruitment criteria, to comprehensive and diversified training on the mental health issues at stake, followed by quality assurance of their performance. Some articles report demanding ways to reach appropriate training: combining video of patients testimonies or doctor-patient interviews, with some immersions into in-patient and ambulatory services, and meeting real patients, in addition to basic SBE training (including learning scenario, readings, in-depth explanations with the trainer, and several behavioural rehearsals (17, 69).

Moreover, ethical issues are raised by the nature of the roles SPs are required to enact. Phenomena such as role adherence, blurring between the role and the person's real life and physical exhaustion are reported (70). The results vary according to different features of the person who depicts the patient: temperament, gender, age, own history and background (and especially psychiatric history, as discussed above). For example, stronger identification effects were reported for adolescents who depicted a psychiatric case, because of contagion effects (71, 72), such as when depicting depression symptoms or suicidal ideations (73, 74). This warrants a careful monitoring of SPs (careful recruitment; proper de-rolling; and close SP monitoring to prevent long-term psychological effects), which may need consideration when allocating resources to SBE.

To address the difficulties of working with trained SPs in psychiatry, the opportunity to recruit real patients appears complex too (75): for example, development of a detached style resistant to any acting and rehearsal training; choice to describe opinions about treatments instead of depicting pre-treatment symptoms. Most of all, playing personal stories for a patient with mental disorders (or a story of their own mental illness) to improve realism creates a risk of potential psychological consequences through to mental health crisis, while for some diseases (such as psychosis), boundaries between thoughts (including delusion) and reality remain blurred. Moreover, possible painful questions raised carelessly during the debriefing may hurt the real patient, even with training. Similarly, the opportunity for a real patient to give appropriate feedback may be further debated given individuals' experience of mental disorders, and the difficulties for patients with mental disorders to adopt enough distance or a metacognitive position to report a more general experience on their pathology. However, if ethical dilemmas and practical issues are given due consideration, involving patients with lived experience in the design and delivery of simulation training can be a very rewarding experience for all involved (76).

## Discussion

SBE appears to be particularly well-suited to psychiatry, supporting a holistic person-centred approach, reflective skills acquisition, emotional elaborations, cognitive reframing and co-construction of care. It also provides an opportunity to involve people with a personal experience of mental disorders in clinical education. However, the validity of the SP portrayal, the complexity of psychotherapeutic skills and the specificities of SPs require due consideration to be effectively implemented.

First, considering the issue of realism of psychiatric portrayal, we should endorse a heuristic notion of “good enough” portrayal rather than “perfect depiction.” Indeed, given the wide variety of singular mental disorders experiences, an excessive essentialisation of symptoms in a unique prototype bares the risk of a robotic portrayal. However, some adjustments may be necessary to reach appropriate learning. Indeed, this notion of “prototype” of psychiatric portrayal may be assumed as a step to make this specialty more accessible to medical students. Moreover, the SP portrayal can be explored during the debriefing with facilitators and observers sharing perspectives from their clinical experience, and by complementary learning activities after SBE (such as real patients video testimonies, workplace supervision of clinical clerkships, among others). For example, even a very short social contact-based video of no more than 90 s can reduce efficiently stigmatisation toward patients with a schizophrenia (77). Furthermore, realism may be fostered by *ad hoc* resources to train SPs. Videos demonstrating good quality portrayals and simulations of people with mental disorders by SPs, based on expert consensus, could provide a digital library to support SP training. This should take into account culturally appropriate portrayals of mental disorder. To reach this consensus and improve the quality and inclusiveness of this library, the group of experts should be multidisciplinary, composed by recognised researchers and experienced practitioners, as well as expert real patients, and medical students. Recent work supports the relevance of skilled video clips of psychiatric SPs depicting psychopathology to teach mental status exam (14), sex education in child and adolescent psychiatry (15), electroconvulsive therapy (16). Furthermore, to prevent a rigid and limited portrayal for each mental disorder, this library may include several variations of a given disorder to cover different clinical presentations. Ultimately, the best way for educators to promote the pedagogy of SBE as a powerful tool against stigmatisation is to be aware, reflexive and constantly collaborating with patients.

Secondly, it appears necessary to acknowledge SBE limitations to train in certain interpersonal skills. For example, the subtle phenomena emerging inside a long-term and familiar psychotherapeutic relationship might not be adequately grasped by SBE training. However, SBE may enable specific training on some specific components of psychotherapeutic skills, counselling skills (78) including some more complex processes such as therapeutic alliance ruptures.

Working on complex interpersonal skills requires deliberate adjustments in the training structure. Indeed, most SBE training assumes that simulated scenarios help to identify participants' performance gap and explore them through facilitated debriefing to close these gaps by highlighting some of the erroneous cognitions leading to these errors. The underlying model of debriefing can be roughly summarised as aiming to enhance clinical performance; in other words a “plus/delta” model of learning. This remains close to the “metaphor of acquisition,” or “the act of gaining knowledge,” which leads most of the literature on learning until the middle of the twentieth century (79). This approach was subsumed over the past decades by the “metaphor of participation,” suggesting that knowledge building is more a result of participation within a group, including being inducted to its language and social rules, through a continuous and collaborative experience rather than transfer or possession of knowledge (79). Yet, in complex interpersonal relationship, such as those within psychiatric care, or also child abuse, end-of-life, there is rarely a single suitable way to behave, in contrast to situations where strong evidence-based guidelines are the rules. Thus, in such complex situations, workers often perceive the same clinical situation differently and agree on a “sensible margin” within which the care must be delivered. Thereby within a constructivist approach, SBE enables for all the participants to observe the same situation, and to share their different perspectives on this same situation during the debriefing, which enables them to learn from each other. The structure of debriefing should thus include the principles of both participation and acquisition, supporting verbalisation of each participant's view, then fostering reflexivity as a group to collaboratively define appropriate behaviours within appropriate care. Due to its familiarity with such complex interpersonal relationships, psychiatric SBE could in turn contribute to all SBE that deals with complex human interactions in medical education.

Similarly, the management of emotions requires dedicated consideration in psychiatry, beyond the attention usually given to emotional responses as part of human factors. Following simulated scenarios, participants are often encouraged to verbalise the elicited emotions. However, this verbalisation often aims to defuse the emotions or recognise its negative impact on performance, to enable the participants to master technical skills. In psychiatric SBE, the identification and elaboration of emotions also aims to regulate emotion so as to improve the participant's self-confidence in care management. However, emotions are also considered as vehicles for meaning and integral to relational experiences that should be entirely integrated in diagnosis and therapeutic practices. As such they remain an important focus of debriefing conversations beyond the initial reaction phase, supporting the deepening of reflection and self-awareness.

Finally, psychiatric SBE remains an essential opportunity to put patients at the centre of medical education. This embodies the health democracy developments of the past decades while supporting a central credo of the Recovery movement: “nothing about us without us” (80). Patients can advise on the co-constructions of appropriate scenarios, using their subjective to supplement professional views, while guiding the acting of SPs through their holistic experiential lens. Their first-person experience can also broaden the reflexivity during debriefing while providing real-life anecdotes which exemplify the importance of words, attention and authenticity of relationships. Their testimony in addition to the affective load mobilised by simulation can support efficiently students' empathy developments accordingly. However, patient involvement must be arranged, structured, and managed carefully and supportively. The fear of retraumatising, symptoms induction, or even inability to fully consent (as in an active psychotic episode) limit the opportunities for them to be SPs and for SBE participation more globally. However, there is limited evidence and guidance on this practice in the literature, meriting further exploration for an area that can have significant benefits.

Recent developments within virtual reality or particular uses of manikins and voice simulations might be a way to circumvent risks of symptoms induction and re-traumatisation. However, for human simulation, clinical educators must be involved in SP recruitment and monitoring to mitigate risks, from the beginning to the end of the training and beyond. This might require clinicians to have a dual role as an educator and manager of the SP pool (including de-rolling after simulations) to retain some clinical insight to assess SPs suitability to remain involved, to contain symptoms emergence, and to ensure adequate follow-up.

The involvement of real patients in SBE presents a wonderful opportunity to hear real patients' voice, and should not be pre-empted by a paternalistic approach that would like to protect patients at their own expense. However, each opportunity to involve patients should not be taken for granted. Any patient involvement needs to be managed with due diligence to individuals' needs, considering both the patient's willingness and the clinician's assessment. Simulated patients are required to enact a complex performance, balancing fidelity with individual learner's reactions and keeping in mind the session learning objectives. These tasks are demanding even for professional actors, and even more so for patients who may not benefit from the professional distance with their own emotional reactions to the subject matter. Patients should retain some freedom to withdraw their involvement every time they can have a role in SBE. The opportunity to engage patients in clinical education should be considered in many creative ways, to contribute to diverse learning experiences, but their utilisation in simulated scenarios requires a very cautious an tentative approach.

## Conclusion

SBE is often seen as a high-tech device, through its heritage rooted in aeronautic or aviation, reflected in its development in technical fields of health care such as anaesthesiology, obstetrics, or surgical specialties. This may scare psychiatric educators away from this training method. However, SBE relates to core dimensions of clinical interactions as multifaceted experiences where communication processes have critical effects, which is particularly true in psychiatry. This can support participants to explore how relationships and communication can have serious consequences for people's health and experience of care. Consequently, SBE is not a mere innovation or by-product of the technological age, but a refined pedagogical approach deeply rooted in a holistic approach as promoted by the World Psychiatric Association (47) in continuity with the medical tradition of a therapeutic *praxis*, as described by Greek philosophers, such as Socrates, and doctors, such as Hippocrates, both emphasising the patient as a whole person and the relationship as an essential conduit of care.

SBE elicits an intense and personal engagement of the learners in a setup borrowing from the performing arts. It offers opportunities for collective reflexivity and co-construction of knowledge that can be compared with community and groups processes as described by anthropologists, systemic approaches, or in mental health institutions. SBE constitutes a rich and flexible cultural artefact lending itself to further creative appropriation, following a diversity of learning needs through many contexts and cultures, present and future.

Finally, clinical educators within psychiatry can greatly benefit from and contribute to the field of SBE, with their clinical experience focusing on intrapersonal, interpersonal, and relational dimensions of care, alongside expert communication skills, that can be transferred from clinical work to the educational setting. We posit that the field of SBE as a whole can benefit from its further implementation and development within mental health education, arguing for clinicians and patients to engage with this powerful pedagogical tool.

## Author Contributions

All authors contributed to the conceptual analysis. M-AP wrote the first draft of the manuscript. The remaining authors commented and modified successive drafts. All authors contributed and have approved the final manuscript.

## Conflict of Interest

The authors declare that the research was conducted in the absence of any commercial or financial relationships that could be construed as a potential conflict of interest.
